# Multi-species probiotics improve growth, intestinal microbiota and morphology of Indian major carp mrigal *Cirrhinus cirrhosus*

**DOI:** 10.1016/j.sjbs.2022.103399

**Published:** 2022-08-01

**Authors:** Md Kabir Hossain, Md Mubarack Hossain, Zabin Tasmin Mim, Habiba Khatun, Muhammad Tofazzal Hossain, Md Shahjahan

**Affiliations:** a Laboratory of Fish Ecophysiology, Department of Fisheries Management, Bangladesh Agricultural University, Mymensingh 2202, Bangladesh; b Department of Fisheries Technology, Bangladesh Agricultural University, Mymensingh 2202, Bangladesh; c Department of Microbiology and Hygiene, Bangladesh Agricultural University, Mymensingh 2202, Bangladesh

**Keywords:** Aquaculture, Bacillus Spp., Lactobacillus spp., Hematology, Gut health

## Abstract

This study aimed to examine the effects of multi-species probiotic on growth, hematological status, intestinal microbes, and intestinal morphology of mrigal (*Cirrhinus cirrhosus*). The mrigal fries (average weight 0.51 g) were reared for 60 days by supplementing multi-species probiotics containing Bacillus spp. (1 × 10^9^ cfu/mL) and Lactobacillus spp. (1 × 10^11^ cfu/mL) in the raising water at doses of 0 (control), 0.5, and 1.0 mL/L. The results indicated that fish reared with multi-species probiotics showed significantly higher growth performance and feed efficiency where the survival rate was similar in all cases. Accordingly, significant higher red blood cell (RBC) and white blood cell (WBC) were counted from the fish reared with multi-species probiotic. There was a considerable difference in bacterial microbiota between the experimental and control group. Multi-species probiotics significantly enhanced the length, width, and villus area. Several immune response indicators like fattening of intestinal mucosal fold, width of lamina propria, the width of enterocytes, and abundance of goblet cells were also increased significantly in fish that received multi-species probiotics. This study revealed that multi-species probiotics can significantly contribute to the growth of mrigal through upgrading intestinal microbiota and morphology, which can be suggested as an eco-friendly growth stimulator in mrigal farming.

## 1 Introduction

Over the few decades, the world population has been increasing sharply, and the world is facing great food security challenges. To meet the world's growing food demand, much effort has been taken into improving aquaculture through both technical and practical approaches (Akter et al., 2021, Teodósio et al., 2020). However, high price and low-quality of feeds, outbreaks of infectious diseases, extensive usage of available chemotherapies, and weakened immune system are the major obstacles for the consistent expansion of aquaculture (Austin and Austin, 2016, Hossain et al., 2020a, Hossain et al., 2020b, Senapin et al., 2018, Uddin et al., 2007). To prevent disease outbreaks, antibiotics and chemicals have been frequently used, which causes resistance to drugs, degradation of the ecosystem, and makes foods unsafe for human health (Aaen et al., 2015, Akanmu, 2018, Dawood and Koshio, 2016). Food safety is a global concern and modern aquaculture producers strive to adopt an aquaculture practice that will be eco-friendly and act as a substitute for antibiotics (Akanmu, 2018, Dimitroglou et al., 2011, Magnadottir, 2010).

A group of beneficial microorganisms is applied in the aquaculture as a sustainable alternative to antibiotics for disease control as well as its nutritional or immune-boosting effects termed probiotics (Lazado and Caipang, 2014, Nayak, 2010, Sharifuzzaman and Austin, 2010). Probiotics are diderm or monoderm bacteria, bacteriophages, algae, and fungi (Austin and Austin, 2016, Soccol et al., 2010, Rohani et al., 2022). Probiotics assist the host or the environment by supporting positive effects that have been isolated from the environment. Terrestrial or aquatic organisms are the bio-friendly components of live microorganisms (He et al., 2017, Rohani et al., 2022). It has been demonstrated that application of probiotics in aquaculture improves the feed efficiency by accelerating digestive enzymes, modulating gut microbiota and prevents various disease by increasing immune-modulatory activities in fish (Elsabagh et al., 2018, Geng et al., 2012, Islam et al., 2021, Jahan et al., 2021). Dietary probiotics increased the growth performance and health condition of the black swordtail, *Xiphophorus helleri* (Hoseinifar et al., 2015) and grass carp, *Ctenopharyngodon idella* (Toutou et al., 2016).

Hematological parameters are considered as an effective index for observing the health status of fish by tracking the changes in fish physiology and pathology and their response to environmental oppression (Michael et al., 2019, Shahjahan et al., 2019, Sharmin et al., 2016). Probiotics have a cabalistic effect on water quality, modification of intestinal microbiota, and enhanced nutrients absorption and metabolism through upgrading the morphology of the gastrointestinal tract (Cerezuela et al., 2012, Devaraja et al., 2013, Han et al., 2015, Zhao et al., 2012). Positive effects of probiotics were observed on the modulation of gastrointestinal microbiota, immune system and disease defense in several cyprinids such as catla Catla catla (Bandyopadhyay and Das Mohapatra, 2009, Das et al., 2013), rohu Labeo rohita (Giri et al., 2012), common carp Cyprinus carpio (Chi et al., 2014) and grass carp Ctenopharyngodon idella (Wang, 2011, Wu et al., 2015). It has been reported that probiotics having the ability to modulate hematological parameters and morphological structure of the intestine, which can be considered an important diagnostic method for monitoring the health condition of cultured fish (He et al., 2011, Michael et al., 2019, Pradhan et al., 2012).

The Indian major carp, mrigal (*Cirrhinus cirrhosus*), is a widely cultivated fish, but recently the production of this species has diminished due to poor aquaculture practices, and low-quality feeds with a high price, and mostly disease outbreaks. In this context, multi-species probiotics can be a promising option for increasing growth and immunity and controlling disease outbreaks (Nayak, 2010, Ramos et al., 2017). Multi-species probiotics (MSP) consist of two or more strains, and several studies revealed that MSP has a more advantageous effect on the host than mono-species probiotics (Lahtinen et al., 2009, Salinas et al., 2008, Wang et al., 2008). In aquaculture, most commonly used multi-species probiotics of bacterial origin including Bacillus spp. and Lactobacillus spp. have been demonstrated significant contributions to growth, feed utilization, defense, and disease resistance in fish (Geng et al., 2012, Giri et al., 2012, Kumar et al., 2008). Although, in the event of carp culture, the application of probiotics has greater potential for improving growth, but single-species probiotics were used in most cases (Kumar et al., 2008). To our best concern, no preceding works have been noticed to assess the impact of multi-species probiotics on growth, hematology, intestinal morphology, and microbiota of C. cirrhosus. Thus, the present investigation was carried out to explore the effects of multi-species probiotics such as Bacillus spp. and Lactobacillus spp. on the growth performance of C. cirrhosus through up gradation of intestinal morphology and microbiota.

## 2 Materials and methods

### 2.1 Experimental fish

The experimental fish were provided from the Hatchery complex, Bangladesh Agricultural University, Mymensingh, Bangladesh. A total of one hundred and eighty healthy, active, uniform size and aged fry were chosen and acclimated in the aquarium at Fish Ecophysiology Laboratory, Bangladesh Agricultural University (BAU), Mymensingh, Bangladesh, for two weeks before feeding the basal diet. The average initial length and weight of fry were 4.1 ± 0.17 cm and 0.51 ± 0.00 g, respectively. During the adaptation and experimental periods, the fry and water quality indicators such as ammonia, pH, and temperature were examined daily. The fries were fed at 9.30–10 a.m. and 5.30–6p.m. and maintained feed at 5 % of their total body weight.

### 2.2 Experimental design

The research was carried with three treatments and each has three replicates for 60 days. 100 L of underground water was used to fill nine aquariums. The volume of each aquarium was (75 × 45 × 45) cm^3^. Multi-species probiotics mixture containing *Bacillus* spp. (1 × 10^9^ colony-forming unit (cfu)/mL) and *Lactobacillus* spp. (1 × 10^11^ cfu/mL) were supplemented with raising water at doses of 0 (control), 0.5, and 1.0 mL/L. The probiotics remaining *Bacillus* spp. were isolated from fish and *Lactobacillus* spp. was derived from yogurt. Twenty fingerlings were stocked randomly in each aquarium after one week of acclimatization and distributed for three treatments with three replications, which lasted for eight weeks. The desired amounts of liquid probiotics were added to the aquarium's rearing water on alternate days during the experiment. When the liquid probiotic was treated in water, it was maintained with extreme caution to avoid cross-contamination. Throughout the study, water quality indicators such as pH, temperature, and ammonia were monitored regularly, and siphoning was done on an opposite day to clean excrement and non-utilized feed and ensured better water quality. Continuous aeration was used to ensure that each aquarium had enough dissolved oxygen (DO) during the experiment. The daily feeding regimen was adjusted every-two weeks based on the fish's weight.

### 2.3 Study of growth, survival, and feed utilization

Individual fish were weighted, and the survival number of fish from each aquarium was documented at the end of the 60 days rearing period. Status of growth and feed utilization such as weight gain= (final weight (g) – initial weight (g)), SGR (specific growth rate) = (ln final weight (g) – ln initial weight (g) / number of days reared × 100) and FCR (Feed conversion ratio) = dry feed fed (g) / live weight (g)) were calculated.

### 2.4 Hemato-biochemical parameters assessment

After 60 days of probiotic treatments, seven fish (n = 7) were sacrificed from each treatment for the collection of the blood sample from the caudal vein region using a micropipette for the measurement of hemoglobin (Hb; g/dL), red blood cell (RBC; ×10^6^/mm^3^), white blood cell (WBC; ×10^3^/mm^3^), and glucose (Glu; mg/dL). A sub-set of blood samples was kept in Eppendorf tubes and added with blood thinners (20 mM EDTA). Each tube was filled up with a 5-μl blood sample for erythrocytes and leukocytes counting. Then preserved blood samples of the erythrocytes and leukocytes were counted using a Neubauer Hemocytometer by adjusted under a light microscope. Another sub-set of blood samples was used to directly determine the Glu and Hb level of Glu and Hb level directly by a digital EasyMate®GHb (Model ET232, Glu/Hb double monitoring system, Bioptic Technology Inc. Taiwan 35,057) with the assist of glucose and hemoglobin strips, separately.

### 2.5 Intestinal microbiota assessment

Total gut microbiota was calculated from each treatment after the experiment was completed. In the case of the intestine, TVC (Total viable count) and LAB (lactic acid bacteria) were determined. Five fish were chosen from each treatment, dissected from the ventral side using sterile scissors, and opened very carefully to separate the intestine from the gastrointestinal tract. The intestinal surfaces were washed with 70 % alcohol and homogenized using a vortex mixer. Single plate-serial dilution spotting (SP-SDS), as described by (Thomas et al., 2015), was used to calculate TVC and LAB. The samples were then serially diluted in sterile saline to a concentration of 10^-8^. For TVC and LAB count, PCA ((Plate Count Agar (Hi media, India)) and MRS agar ((De Man, Rogosa, and Sharpe (Hi media, India)) were used. One to eight dilution parts were marked with a marker on the surface of the Petri-dishes for Total viable and Lactic acid bacteria count. With the help of an adjusted micropipette, two drops of 10 μL aliquant from each dilution were used to each part and allowed for drying in a streamlined flow cabinet. For TVC, the plates were placed at 37 °C in the incubator for 24 h. For LAB, the plates were also kept in an anaerobic candle jar and allowed at 37 °C for 72 h for incubation. Colony-forming unit/g was assessed after 12 h incubation for TVC and 48 h for LAB by pointing the colonies on the other side of the plate.

### 2.6 Histological evaluation of the intestine

After the experiment was completed, the three sampled fish individuals from each treatment were dissected from the ventral side and opened extra carefully to remove the intestine from the gastrointestinal tract. Undesirable components were removed from the intestine and preserved for 24 h in marked vials containing Bouin's solution, then transferred to 70 % alcohol for storage and maintained at 4 °C prior to histological analysis.

A series of alcohol treatments (sink in 70 %, 80 %, 90 % and 95 % ethanol for 24, 12, 1 and 1 h, respectively, and finally trice in 100 % ethanol for 40 min) were carried out as a dehydration process for preserved intestinal tissues. Then, the oven was used for infiltration for an hour in molten paraffin wax. Infiltrated tissues were embedded in paraffin wax and maintained in a cold basin. The pieces were cut at 5 μm thicknesses through a microtome machine after trimming from prepared blocks. Sections were placed on a glass slide and left to air dry overnight. Two time-xylene treatments were performed for 20 min to eliminate the paraffin wax from the whole slides and allowed a series of alcohol treatments in descending sequence. Hematoxylin-Eosin solution was used to stain the sectioned tissues in the end.

Morphological parameters of the intestine were examined using an electric microscope (MCX100, Micros Austria). Morphology such as crypt depth (µm), villus length (µm), villus width (µm), villus area (mm^2^), the thickness of the gut wall (µm), width of lamina propria, abundance of goblet cell (GB), enterocyte width (EC), fattening of the mucosal fold (μm) was assessed by applying image analysis software (Sigma Scan Pro5, SPSS Inc) as mentioned by Bullerwell et al. (2016).

### 2.7 Statistical analysis

Data was collected, documented, and saved on a computer spreadsheet for statistical analysis during the experiment. The statistical variance was calculated among the treatment groups using a one-way analysis of variance (p < 0.05). The mean, as well as standard deviation, were used to depict all of the data (SD). Image processing analytical software was used to perform morphological evaluations of the intestine. All analytical data were calculated using the PASW Statistics 18.0 program (IBM SPSS Statistics, IBM, Chicago, USA).

## 3 Results

### 3.1 Growth status and feed utilization

The effects of multi-species probiotics on growth and feed utilization indices such as weight gain (WG), SGR, FCR and survivability of C. cirrhosus are presented in Table 1. After 60 days of the investigational period, it was noticed that growth indicators were considerably different (p < 0.05) in those groups that received multi-species probiotics in contrast with the control group (no probiotics). The best performances in the case of final weight gain and SGR were obtained from the fish reared with 1.0 mL/L of multi-species probiotics compared to the fish reared with 0.5 and 0 mL/L of probiotics, respectively (Table 1). A significantly lower (p < 0.05) FCR was also obtained from the fish group reared with 1.0 mL/L of multi-species probiotics, where the highest was obtained from the control (Table 1). For all the treatments, the feeding trials did not affect the fish survivability.

**Table 1:** Growth performance of Indian major carp mrigal reared with multi-species probiotics for 60 days.

Parameters	Addition of probiotics (ml/L)		
	0	0.5	1.0
Initial body weight (g)	0.50 ± 0.02	0.49 ± 0.04	0.49 ± 0.01
Final body weight (g)	2.54 ± 0.25^a^	3.34 ± 1.59^ab^	4.05 ± 0.42^b^
Weight gain (g)	2.04 ± 0.25^a^	2.85 ± 0.49^ab^	3.56 ± 0.42^b^
Specific growth rate (SGR; %/day)	0.73 ± 1.06^a^	0.94 ± 0.36^ab^	1.08 ± 0.67^b^
Feed conversion ratio (FCR)	1.98 ± 0.25^b^	1.44 ± 0.25^ab^	1.13 ± 0.11^a^
Survival (%)	100.00 ± 0.00	100.00 ± 0.00	100.00 ± 0.00

All values are presented as mean ± SD. The alphabetical superscripts in the values indicate significantly (p < 0.05) different among different treatments in each row.

### 3.2 Hematological parameters

The hematological parameters such as Hb, RBC, WBC, and Glu of C. cirrhosus reared with different levels of multi-species probiotic were gathered, and the outputs were concised in Table 2. In the case of mean RBC and WBC counts, significantly (p < 0.05) highest number were recorded for the fish group reared with 1.0 mL/L multispecies probiotics (Table 2). But, insignificant differences (p < 0.05) in Hb were observed among the collections where the highest value was documented from the control group. However, in the case of glucose level, the lowest value was recorded from the fish group reared with 0.5 mL/L of multi-species probiotics than the others with no significant (p < 0.05) difference was discerned among the treatments (Table 2).

**Table 2:** Alterations of hemato-biochemical parameters of Indian major carp mrigal reared with multi-species probiotics for 60 days.

Parameters	Addition of probiotics (ml/L)		
	0	0.5	1.0
Hb (g/dL)	13.56 ± 1.10	13.24 ± 3.25	12.90 ± 0.33
RBC (×10^6^/mm^3^)	0.98 ± 0.24^a^	1.35 ± 0.34^ab^	1.60 ± 0.63^b^
WBC (×10^3^/mm^3^)	1.02 ± 0.42^a^	1.58 ± 0.26^ab^	2.05 ± 0.47^b^
Glucose (mg/dL)	144 ± 28.64	118.8 ± 25.81	127.11 ± 10.61

Hb; hemoglobin, RBC; red blood cell, and WBC; white blood cell. All values are presented as mean ± SD. The alphabetical superscripts in the values indicate significantly (p < 0.05) different among different treatments in each row.

### 3.3 Changes in intestinal microbiota

For observing the effect of multi-species probiotics on the gut microbiota of *C. cirrhosis*, TVC and LAB were determined, and the results are shown in Table 3. As shown in Table 3, after 60 days of the rearing period, all the probiotic supplemented fish groups were exposed to a significantly(p < 0.05) higher number of total viable count and lactic acid bacteria in the gut than the control group. The highest TVC was recorded from the fish group reared with 0.5 mL/L of multi-species probiotics, while the fish reared with 1.0 mL/L of multi-species probiotics showed the highest load of LAB than the rest of the treatments.

**Table 3:** Total viable count (TVC) and lactic acid bacteria (LAB) in the gut of Indian major carp mrigal reared with multi-species probiotics for 60 days.

Parameters	Addition of probiotics (ml/L)		
	0	0.5	1.0
TVC (×10^5^ cfu g^−1^ of gut)	0.003 ± 0.001^a^	45.000 ± 5.000^c^	11.167 ± 3. 4037^b^
LAB (×10^2^ cfu g^−1^ of gut)	0.27 ± 0.10^a^	6.25 ± 1.77^b^	11.00 ± 1.00^c^

All values are presented as mean ± SD. The alphabetical superscripts in the values indicate significantly (p < 0.05) different among different treatments in each row.

### 3.4 Changes in intestinal histomorphology

**Fig. 1:**
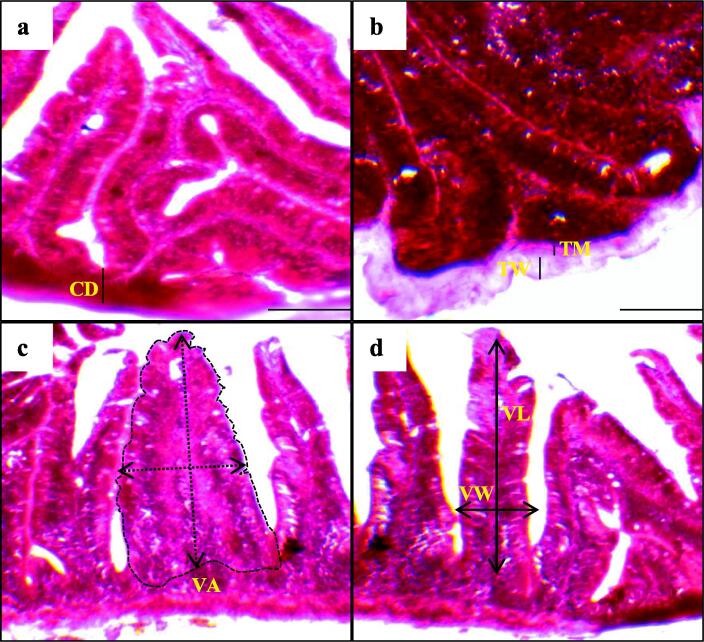
Histological alteration of the intestine of Indian major carp mrigal (*Cirrhinus cirrhosus*) reared with multi-species probiotics for 60 days; (a) control (0 mL/L), (b) low dose (0.5 mL/L), (c, d) high dose (1.0 mL/L); VW; villus width, VL; villus length, VA; villus area, TW; thickness of wall, TM; thickness of muscular, and CD; Crypt depth. Scale bar = 100 µm.

**Fig. 2:**
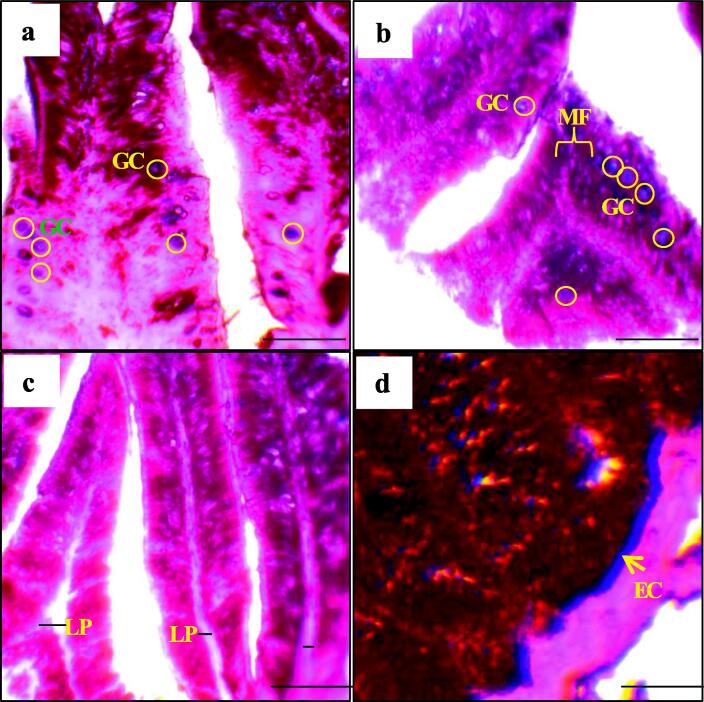
Immune response indicators (a-d) of histological gut of Indian major carp mrigal (*Cirrhinus cirrhosus*) reared with multi-species probiotics for 60 days; MF; mucosal folds, LP; lamina propria, GC; goblet cell and EC; enterocyte. Scale bar = 400 µm.

The consequence of multi-species probiotics on different intestinal morphometric characteristics of C. cirrhosus like crypt depth (µm), villus length (µm), villus width (µm), villus area (mm^2^), the thickness of the intestinal wall (µm), and the thickness of muscular (µm) were examined by an electronic microscope. The findings are presented in Table 4 and Fig. 1. At the completion of the trial, it was noticed that the fish reared with 1.0 mL/L of multi-species probiotics indicated significantly higher (p < 0.05) measurements of the wall thickness, muscular thickness, villus length, width, areas, and crypt depth of the intestine compared to the other fish groups where those above values ranged from 296 to 358 µm, 110 to 162 µm, 32 to 58 mm^2^, 14 to 38 µm, 8 to 19 µm and 49 to 102 µm, respectively (Table 4). In addition, changes in various immune response indicators of the histological gut such as the width of lamina propria, goblet cell, enterocytes width, and mucosal fold result from rearing fish with multi-species probiotics are presented in Table 5 and Fig. 2. Finally, it was found that the above mentioned immune response indicators were significantly (p < 0.05) different among the groups, and the maximum values were observed from the fish group reared with 1 mL/L of multi-species probiotics compared to 0.5 and 0 mL/L (control) of probiotics, respectively,

**Table 4:** Gut morphology of Indian major carp mrigal reared with multi-species probiotics for 60 days.

Parameters	Addition of probiotics (ml/L)		
	0	0.5	1.0
Villus width (μm)	110.50 ± 3.89^a^	139.75 ± 6.61^ab^	162.50 ± 7.43^b^
Villus length (μm)	296.25 ± 7.57^a^	319.25 ± 3.33^ab^	358.50 ± 6.12^b^
Villus area (mm^2^)	32.73 ± 1.40^a^	44.62 ± 2.45^ab^	58.27 ± 3.40^b^
Thickness of wall (μm)	14.50 ± 1.10^a^	25.75 ± 2.05^ab^	38.50 ± 1.10^b^
Thickness of muscular (μm)	8.50 ± 1.10^a^	14.25 ± 1.16^ab^	19.75 ± 0.87^b^
Crypt depth (μm)	49.75 ± 1.58^a^	63.75 ± 2.43^ab^	102.50 ± 1.93^b^

All values are presented as mean ± SD. The alphabetical superscripts in the values indicate significantly (p < 0.05) different among different treatments in each row.

**Table 5:** Immune response indicators of histological gut of Indian major carp mrigal reared with multi-species probiotics for 60 days.

Parameters	Addition of probiotics (ml/L)		
	0	0.5	1.0
Width of lamina propria (μm)	6.50 ± 1.29^a^	13.75 ± 1.71^b^	23.75 ± 2.75^b^
Abundance of Goblet cell (GB)	53.00 ± 6.19^a^	72.25 ± 6.63^ab^	96.75 ± 6.02^b^
Enterocyte width (μm)	3.50 ± 0.58^a^	6.33 ± 0.58^b^	7.75 ± 0.89^b^
Fattening of mucosal fold (μm)	8.00 ± 0.76^a^	11.50 ± 1.10^ab^	17.00 ± 0.76^b^

All values are presented as mean ± SD. The alphabetical superscripts in the values indicate significantly (p < 0.05) different among different treatments in each row.

## 4 Discussion

The multi-species probiotics are recognized to improve the growth performance of fish and shellfish species through modification of microbial community, exclusions of the pathogen, up-gradation of a non-specific immune response, and disease resistance (Li et al., 2009, Qi et al., 2009, Rodiles et al., 2018, Sun et al., 2010). Among probiotics, *Bacillus* spp. and L*actobacillus* spp. have become more popular and widely used in aquaculture (Geng et al., 2012, Harikrishnan et al., 2010). In our study, *C. cirrhosis* fries were reared with three different concentrations of multi-species probiotics for 60 days which resulted in a substantial increase in final weight gain and specific growth rate among the treatments where better FCR was obtained from the fish reared with 1.0 mL/L of multi-species probiotics compared to 0.5 and 0 mL/L of probiotics, respectively. A considerable amelioration in growth factors of *L. rohita* was noticed reared with *Bacillus* spp. (Giri et al., 2014). It has been reported that probiotics (*Bacillus* spp.) supplementation significantly increased the weight and SGR in the Nile tilapia, *Oreochromis niloticus* (Zhou et al., 2010), rainbow trout, *Oncorhynchus mykiss* (Merrifield et al., 2010), yellow croaker, *Larimichthys crocea* (Ai et al., 2011), grouper, *Epinephelus coioides* (Sun et al., 2010) and cobia, *Rachycentron canadum* (Geng et al., 2012)*.* Besides, Carnevali et al., 2004, Kesarcodi-Watson et al., 2008 witnessed that the growth and survival rate of the farmed rainbow trout and sea bream increased significantly due to the administration of *Lactobacillus* spp. as probiotics. It has also been reported that probiotic mixtures (*Bacillus* spp. and *Lactobacillus* spp.) triggered the growth performance of fish (Ramos et al., 2017, Salam et al., 2021. This might be possible as they could improve digestion and nutrient absorption, stimulating extracellular enzymes such as amylases, proteases lipases and influenced on their intestinal morphology (Ramos et al., 2017, Karthik et al., 2018). Moreover, the enzymatic activities could be a reason for better growth performance and health status of fish because diverse proteins are usually broken down into their constituent amino acids by bacterial proteases that ultimately provide much nutrition to the fish (Ran et al., 2012). In addition, the administration of probiotics like *Bacillus* spp. and *Lactobacillus* spp. to the fish significantly stimulates appetite and enhances microbial metabolism (Irianto and Austin, 2002). Through increasing digestibility and absorption of swallowed food, probiotics enhance feeding efficiencies that lead to the high growth of fish (Balcázar et al., 2006). On the other side, probiotics changed the microbial community of the fish gut through active competition with other bacteria for space, nutrition and excluded harmful bacteria by the production or secretion of compounds like bacteriocins (Kesarcodi-Watson et al., 2008). They also ensured the availability of numerous growth factors like fatty acids, essential amino acids, and vitamins K and B_12_ which enhance the growth of fish (Ray et al., 2012, Sumon et al., 2018). Moreover, multi-species probiotics play a vital role in improving FCR. Yanbo and Zirong (2006) noticed a better FCR for common carp reared with multi-species probiotics than controls, and similar findings were also observed from the current study which indicates the effectiveness of multispecies probiotics in fish growth.

In the current study, a significant increase in WBC and RBC count was observed from fish reared with multi-species probiotics. Hematological characteristics are thought to be a good indicator of fish health (Shahjahan et al., 2022). The effects of probiotics on hematological parameters of different species of fish have been studied by several authors (Jahan et al., 2021, Korkea-aho et al., 2012, Salam et al., 2021, Soltani et al., 2019, Talpur et al., 2014) who informed that mixture of probiotics such as *Bacillus* spp., *Lactobacillus* spp. significantly increased the number of RBC and WBC in fish, which confirms the current study's findings. Moreover, probiotics release organic acids in the gut, enhancing iron absorption and facilitate the multiplication of RBC, lymphocytes, granulocytes, and macrophages in fish, similar to higher her vertebrates (Kumar et al., 2008, Yadav et al., 2007, Dahiya et al., 2012). An interaction between probiotics and immune cells like neutrophils, monocytes, macrophages, and lymphocytes has been noticed by Nayak, 2010, Rajikkannu et al., 2015 which improves innate immune response. On the other hand, WBC plays a significant role in strengthening fish’s efficiency in fighting against infectious microorganisms (Weiss and Wardrop, 2011). In our present study, fish reared with multi-species probiotics showed more WBC than the other fish groups, suggesting that mixed probiotics could induce leucocyte production in fish. It serves as a protective barrier against pathogens (Talpur et al., 2013) and engages in altering innate immunity through phagocytosis and toxic cell formation (Chico et al., 2018, Puente-Marin et al., 2019). Therefore, it is assumed that probiotics could increase immunity in the fish (Panigrahi et al., 2005). On the other hand, catecholamine stimulated hyperglycemia in fish, resulting from a higher blood glucose level which is an sign of physiological stress in fish (Shahjahan et al., 2020, Silva et al., 2015, Simões et al., 2012, Vhatkar et al., 2016). In our study, there was no distinct change in blood glucose levels with multi-species probiotics, which indicates that fish reared with multi-species probiotics are healthier. In addition, through stimulating lymphocyte proliferation (both B and T cells), probiotics represent the immune-stimulant and anti-infective properties responsible for upgrading immunoglobulin and antibody levels that may positively influence the health status of fish (Al-Dohail et al., 2009, Picchietti et al., 2009).

Generally, the gut microbiota is related to digestion, metabolism, and absorption of nutrients in the host (Adeoye et al., 2016, Yu et al., 2019). The current study expressed that after the 60 days of the rearing period, supplementation of multi-species probiotics significantly increased the TVC and LAB in the gut of *C.* cirrhosis than the control group (Table 3). This could be due to the more proclivity of colonization of Bacillus spp. and Lactobacillus spp. in the intestine, which also prevents the multiplication of pathogenic bacteria. Several authors reported that Bacillus spp. and Lactobacillus spp. improved the bacterial community in the intestine of aquatic organisms, especially in fish, by destroying harmful bacteria and facilitating the colonization of beneficial bacteria (Elsabagh et al., 2018, Hlordzi et al., 2020, Opiyo et al., 2019). Furthermore, Opiyo et al. (2019) reported that TVC (total plate count) of gut bacteria improved due to the usage of Bacillus spp. in O. niloticus and the application of Lactobacillus spp. enhanced the intestinal LAB (lactic acid bacteria) for common carp (Alishahi et al., 2018, Yu et al., 2019), which supports the findings of the existing study.

The physiology and metabolism of aquatic animals can be affected by gastrointestinal morphology. It has been stated that probiotics improve the intestinal histo-morphometric characteristics of fish by changing the intestine's structure and cellular renewal (Cerezuela et al., 2013, Hisano et al., 2018, Schwarz et al., 2011). In the present study, it was noticed that multi-species probiotics significantly changed the intestinal morphology through improving the thickness of the wall, muscular layer, length, width, and area of the gut villus, as well as influenced the mucosal fold and some immunological responses than the control (Table 4, Fig. 1) which subsequently indicates a healthy intestine may be due to the combine effects of both *Bacillus* spp. and *Lactobacillus* spp. (Khojasteh, 2012). Similarly, *Bacillus* spp. significantly influenced the area of nutrient absorption, retention, villi length, goblet cells count, and enterocyte height of the intestine of different fish species (Elsabagh et al., 2018, Mello et al., 2013, Silva et al., 2015). The enhancement of the length, width, area, and thickness of the intestinal villi, which are evaginations of the intestinal mucosa, is strictly linked to the absorption of intestinal nutrients and contributes to utilization of feed and growth status of fish (Ferguson et al., 2010, Khojasteh, 2012, Pirarat et al., 2011, Schwarz et al., 2011). In our study, along with the changes in intestinal morphology, a significant difference in several immune response indicators such as lamina propria, enterocyte width, goblet cells and intestinal mucosal fold were experienced with increasing the concentration of multi-species probiotics (Table 5 and Fig. 2). In covenant with the outcomes of the current study, Abdel-Aziz et al., 2020, Pirarat et al., 2011, Schwarz et al., 2011, Standen et al., 2016 reported that probiotics increased the goblet cell count in fish. Goblet cells produce bactericidal mucus that aids in the trapping and removal of infections, combats harmful substances, reduces dehydration, and serves as a protective barrier for the gastro-intestine (Ferguson et al., 2010, Whyte, 2007). On the other hand, the passage of nutrients is regulated by enterocytes, which act as absorptive cells (Cerezuela et al., 2012, Khan and Islam, 2012). In addition, mucin components such as mucopolysaccharides and glycoproteins are contained by mucus involved in pathogen antagonism and ultimately play vital roles in improving the health of fish (Liao and Nyachoti, 2017, McGuckin et al., 2011).

Overall multi-species probiotics administration in water showed positive effects on feed utilization, improvement of hematological parameters, up gradation of intestinal microbiota and morphology, which significantly enhanced the growth of the C. cirrhosus. However, further study is required to see the different enzymatic activity and disease resistance occurred due to probiotics on host species. It is also necessary to examine which bacterial strains are responsible for specific organ development.

## Declaration of Competing Interest

The authors declare that they have no known competing financial interests or personal relationships that could have appeared to influence the work reported in this paper.
